# Biallelic Variants in *CCDC39* Gene Lead to Primary Ciliary Dyskinesia and Kartagener Syndrome

**DOI:** 10.1155/2022/7130555

**Published:** 2022-06-26

**Authors:** Xiao Shi, Hao Geng, Hui Yu, Xiaolong Hu, Guanxiong Wang, Jin Yang, Hui Zhao

**Affiliations:** ^1^Department of Respiratory and Critical Care Medicine, The Second Affiliated Hospital of Anhui Medical University, Hefei, China; ^2^Reproductive Medicine Center, Department of Obstetrics and Gynecology, The First Affiliated Hospital of Anhui Medical University, Hefei, China

## Abstract

**Background:**

Primary ciliary dyskinesia (PCD) is a clinical syndrome characterized by cilia with an abnormal structure or function. Its main clinical manifestations comprise chronic bronchitis, cough, recurrent respiratory infections, situs inversus, and male infertility. Single-gene variants are widely assumed to be the main cause of this rare disease, and more than 40 genes have been described to be associated with its onset. *CCDC39* is essential for assembling the inner dynein arms and dynein regulatory complex and is important in cilia motility. *CCDC39* variants were reported as a monogenic etiology of PCD.

**Methods:**

This study investigated two unrelated Chinese patients diagnosed as PCD. The chest computed tomography scan was performed to identify PCD phenotypes of the two probands. Considering the effect of PCD on male fertility, routine semen analysis, sperm morphology examination, and scanning electron microscopy were performed to assess the semen characteristics of male proband in family 2 (F2 II-1), who had a history of infertility. Subsequently, the peripheral blood samples of probands were collected to perform whole-exome sequencing (WES) to explore the possible genetic causes of this disease.

**Results:**

Whole-exome sequencing revealed a homozygous *CCDC39* variant in the female proband of family 1 (F1 II-1: c.286C>T:p.Arg96Ter) and two compound heterozygous *CCDC39* variants in the male proband of family 2 (F2 II-1: c.732_733del: p.Ala245PhefsTer18; c.2800_2802dup:p.Val934dup). The two probands showed the typical PCD phenotypes, including chronic bronchitis, recurrent respiratory infections, and situs inversus. The male proband also showed oligoasthenoteratospermia with multiple morphological abnormalities of the sperm flagella. Additionally, CCDC39 protein level was significantly lower in the sperm of male proband than in the sperm from normal controls.

**Conclusion:**

We identified a homozygous variant reported previously and two compound heterozygous variants of *CCDC39* possibly responsible for PCD pathogenesis, expanding the variant spectrum of Chinese PCD, Kartagener syndrome, and morphological abnormalities of the sperm flagella involving *CCDC39*.

## 1. Introduction

Primary ciliary dyskinesia (PCD, OMIM#244400) is a rare and recessively inherited disorder caused by defects in the structure or function of cilia (1–3). Approximately 1 in 10,000–20,000 individuals suffer from this syndrome worldwide, and the prevalence of PCD is high in highly consanguineous communities (2, 4, 5). Because of the wide distribution of cilia, PCD can involve multiple organs or systems and greatly impacts the patients' quality of life (6). PCD most frequently affects the respiratory system, which is characterized by recurrent respiratory tract infections, chronic wet cough, and bronchiectasis, among other effects. PCD in other systems can manifest clinically with diverse symptoms such as otitis media, hearing defects, rhinitis, sinusitis, and infertility (7–9). Neonatal respiratory distress syndrome is a phenotype of PCD occurring in early life (10, 11). Additionally, defective cilia in developing embryos may lead to situs inversus, resulting in Kartagener syndrome, in approximately half of patients with PCD (12–14).

More than 40 genes have been shown to be associated with the disease onset (2). Single-gene variants are widely considered to account for 70–80% cases of PCD (15). Proteins encoded by these genes are involved in cytoplasmic preassembly axonemal dynein and the assembly of outer dynein arms (ODAs), inner dynein arms (IDAs), and radial spokes, among other processes (2). Dynein axonemal assembly factors are mainly responsible for preassembling ODAs and IDAs in the cytoplasm before these proteins reach their final docking sites (16). Variants in the genes encoding ODAs and IDAs can cause defects in these proteins, leading to PCD development (2). For instance, in 1999, as a member of the dynein axonemal assembly factors, *DNAI1* (OMIM: 604366) was the first gene shown to be involved in PCD and numerous studies have substantiated the association between PCD and variants in this gene (17, 18). ODAs play an important role in enabling cilia bending. Variants in the genes encoding components of ODAs are also common causes of PCD, such as variants of *DNAH5* (OMIM: 603335) and *DNAH11* (OMIM: 603339) (19, 20). Additionally, *CCDC39* (OMIM: 613798) and *CCDC40* (OMIM: 613799) are essential for the assembly of IDAs and the dynein regulatory complex, as well as for the correct establishment of 96 nm repeats along the ciliary axoneme. Both are also causative genes of PCD (21, 22).

In this study, whole-exome sequencing (WES) was performed in two unrelated Chinese individuals with PCD and Kartagener syndrome to detect the possible genetic cause. And we also assessed the semen characteristics of the male proband using routine semen analysis, sperm morphology examination, and scanning electron microscopy. A homozygous variant reported previously and two compound heterozygous variants were identified. In addition, the male proband showed oligoasthenoteratospermia with multiple morphological abnormalities of the sperm flagella (MMAF).

## 2. Subjects and Methods

### 2.1. Participants

Two Han Chinese patients (F1 II-1 and F2 II-1) clinically diagnosed as PCD and Kartagener syndrome were recruited from the Second Affiliated Hospital of Anhui Medical University (Hefei, China) and First Affiliated Hospital of Anhui Medical University (Hefei, China), respectively. The female proband (F1 II-1) was from a consanguineous family, whereas the male proband (F2 II-1) was not. Whole-exome sequencing was performed using the whole peripheral blood samples collected from the two probands. This research was approved by the Ethics Committee of the Anhui Medical University, and all patients and their relatives agreed to participate in the study and provided written informed consent. Two fertile male participants with normal sperm density, motility, and morphology were also recruited as controls, and no significant PCD-related clinical manifestations were detected.

### 2.2. WES and Sanger Sequencing

Genomic DNA was extracted from the whole-peripheral blood samples from F1 II-1 and F2 II-1. The whole exome was sequenced on the Illumina HiSeq platform (San Diego, CA, USA). The original FASTQ data were mapped to the human genome using the BWA software, and SAM tools and GATK were used to call genetic variants. We annotated variants using the allele frequency database (1000G, ExAC_all, and gnomAD), deleterious prediction tools (SIFT, PolyPhen-2, and MutationTaster), and Human Gene Mutation Database using ANNOVAR (23) and dbNSFP (24). Common variants with an allele frequency of >0.05 were excluded. We focused on loss-of-function (including splicing (≤2 bp), stop-gain, stop-loss, and frameshift indels) and deleterious missense variants. Variants predicted to be deleterious by three software programs, namely, SIFT, PolyPhen-2, and MutationTaster, were included for further evaluation. Moreover, variants defined as deleterious in the Human Gene Mutation Database were also included in the analysis. Sanger sequencing was performed to validate the identified variants in the probands and their available family members to verify the inheritance pattern.

### 2.3. Semen Analysis

Based on World Health Organization guidelines (5th edition), the semen samples from F2 II-1 were subjected to sperm routine analysis and sperm morphology examination. Semen samples were collected following 3–7 days of abstinence and evaluated after liquefaction for 30 min at 37°C (25).

### 2.4. Western Blotting

To determine the expressive level of CCDC39 in the spermatozoa from F2 II-1, western blotting was performed as described in a previous study (26). In brief, the spermatozoa protein was separated by SDS-PAGE and then transferred onto a PVDF membrane (Millipore, Burlington, MA). Subsequently, the membrane was blocked with 5% defatted milk and diluted with TBST. After being incubated with primary antibodies at 4°C overnight and then incubated with secondary antibodies at 37°C for 2 hours, the protein was detected by enhanced chemiluminescence reagents (Thermo Scientific). The primary antibodies used for western blotting were anti-CCDC39 antibody (1 : 1000, HPA035364, Atlas Antibodies, Bromma, Sweden) and anti-beta actin antibody (1 : 8000, ab8224, Abcam, Cambridge, UK).

### 2.5. Scanning Electron Microscopy

A part of the sperm sample from F2 II-1 was used for scanning electron microscopy according to a previously described protocol (27). The fresh sperm samples were centrifuged and washed twice. Subsequently, the spermatozoa were deposited on poly-L-lysine-coated coverslips, immersed in 2.5% phosphate-buffered glutaraldehyde, and postfixed in osmic acid. After being rinsed thoroughly in phosphate buffer and dehydrated in a series of ethanol dilutions at increasing concentrations, the processed semen specimen was placed on coverslips to dry naturally. Afterwards, the sperm samples were coated with gold particles and then visualized with an S-3400N scanning electron microscope (Hitachi, Tokyo, Japan) under an accelerating voltage of 20 kV.

## 3. Results

### 3.1. Clinical Characteristics and Sperm Analysis of the Two PCD Patients

**Figure 1:**
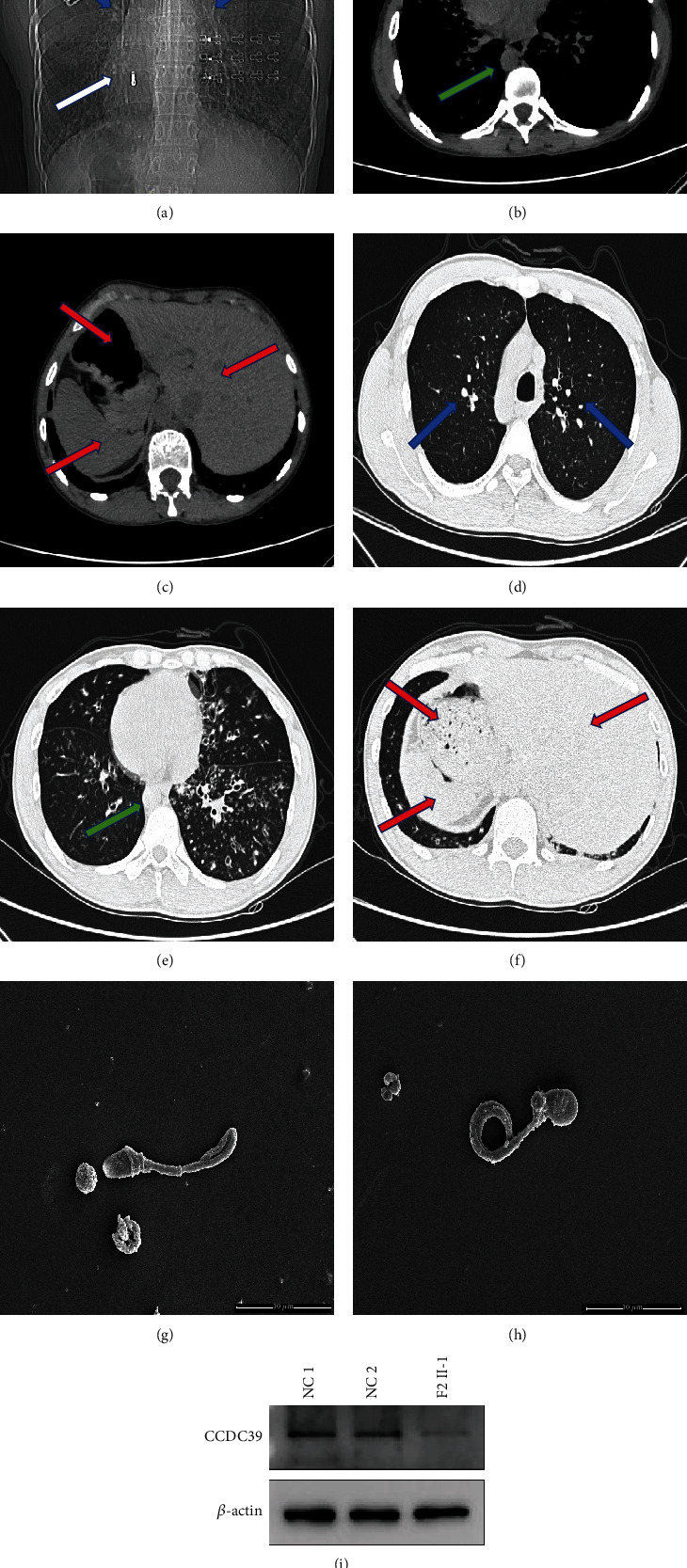
Clinical characteristics of two PCD patients. (a–c) Chest CT scanning of F1 II-1. (d–f) Chest CT scanning of F2 II-1. Blue arrows: chronic inflammatory changes and bronchiectasis of the lung lobes. White arrow: typical cardiac inversion. Green arrows: aortic arch is located on the right side of the chest cavity. Red arrows: ectopic internal organs (liver, stomach, and spleen). Scanning electron micrograph of spermatozoa from F2 II-1 indicates the MMAF phenotype, including (g) short and (h) coiled sperm flagella (scale bars, 10 *μ*m). (i) The results of western blotting show decreased expression level of CCDC39 protein in the spermatozoa of F2 II-1. NC: normal control.

Two unrelated Han Chinese (F1 II-1 and F2 II-1) were recruited based on their canonical PCD phenotype, such as chronic bronchitis, recurrent respiratory infections, and situs inversus. F1 II-1, the female PCD patient from a consanguineous family, suffered from recurrent infections of the airways and chronic wet cough for 10 years. Chest computed tomography (CT) scan indicated typical PCD phenotypes. F2 II-1, the male patient who shared the same PCD-related phenotype with F1 II-1, had been diagnosed with infertility two years prior. Chest computed tomography imaging corroborated the clinical manifestations of PCD (bronchiectasis and situs inversus) in the two probands. No PCD-related clinical manifestation was observed in other available members of the two families (F1 I-1, F1 I-2, F1 II-2, F2 I-1, and F2 I-2). Semen analyses of F1 II-1 revealed severe oligoasthenozoospermia, and nearly all spermatozoa were immotile. Subsequent sperm morphology examination and scanning electron microscopy showed that most spermatozoa had a high percentage of multiple flagellar malformations, including short, coiled, and irregular flagella. Detailed information is described in Table 1 and Figures 1(a)–1(h).

### 3.2. Identification of Biallelic CCDC39 Variants in PCD Patients

**Figure 2:**
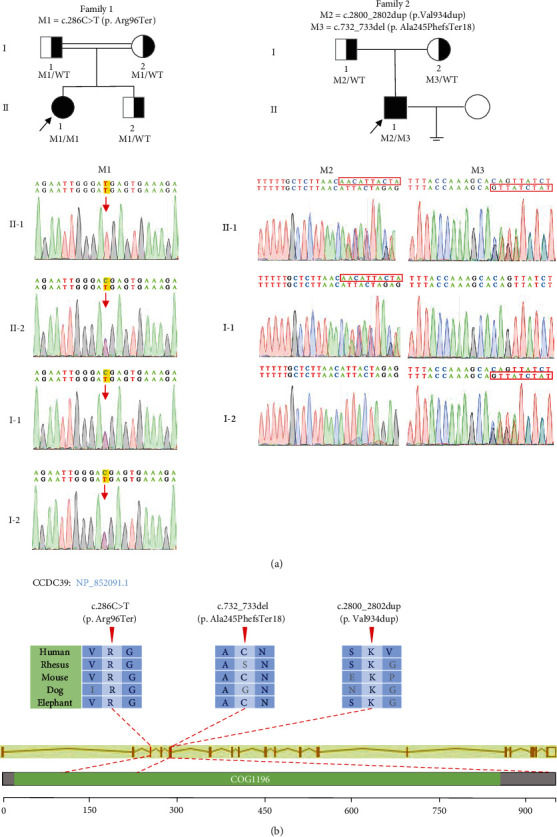
Identification of biallelic variants of *CCDC39* in two PCD patients. (a) Pedigree and genotypes of two PCD family members carrying *CCDC39* variants. The probands are marked with black arrows. The red arrow and dashed boxes indicate mutated locations in the Sanger sequencing results. (b) A schematic diagram of mutated positions that occurred in the genomic and protein structure of CCDC39. The red arrows indicate the locations of *CCDC39* variants occurred in the domain of CCDC39 protein. WT: wild type; M: *CCDC39* variant.

To explore the genetic cause of PCD, whole-exome sequencing and bioinformatic analyses were performed on the two PCD patients, leading to the identification of three variants of *CCDC39*. No other PCD-associated genes were found. These variants were verified using Sanger sequencing and are illustrated in Figure 2. In consanguineous family 1, a homozygous stop-gain variant (NM_181426:c.286C>T:p.Arg96Ter) in *CCDC39* was identified in F1 II-1, which was inherited from his heterozygous parental carrier. Her brother (F1 II-2) was a heterozygous carrier of this variant and showed normal semen parameters without PCD-related clinical manifestation. In the unrelated family 2, two compound heterozygous variants (NM_181426:c.732_733del: p.Ala245PhefsTer18; c.2800_2802dup:p.Val934dup) of *CCDC39* were identified in F2 II-1 and validated as inherited from his parental carriers. All of these variants are rare or absent in the 1000 Genomes Project, ExAC, and GnomAD databases (Table 2).

### 3.3. Expression Level of CCDC39 Protein in the Spermatozoa of F2 II-1

To further investigate the pathogenicity of the identified *CCDC39* variants at the protein level, spermatozoa were collected from F2 II-1 for western blotting. Compared to that in the normal control, the expression level of CCDC39 protein in the spermatozoa of F2 II-1 was significantly decreased (Figure 1(i)).

## 4. Discussion

*CCDC39*, localized on chromosome 3q26.33, encodes a core axonemal protein that participates in the assembly of IDAs and the dynein regulatory complex and plays a critical role in establishing the 96 nm repeats along the ciliary axoneme combined with *CCDC40* (2). Ultrastructurally, CCDC39-mutated cilia commonly present defects in IDAs, the dynein regulatory complex, and radial spokes, with normal ODAs (2). Variants in this gene can cause defective cilia beat regulation and ciliary immobility (21). As one of more than 40 genes responsible for PCD, *CCDC39*-related PCD is inherited in an autosomal recessive pattern, and patients with *CCDC39* variants commonly suffer from sinusitis, bronchitis, bronchiectasis, laterality defects, and infertility, among other conditions (2, 21). Numerous studies have shown that variants in this gene cause PCD in patients from different geographic locations and diverse ethnic groups (10, 21, 28–30). In 2010, Merveille et al. firstly positionally cloned *CCDC39* and reported an association between PCD and this cilia-related gene in dogs and humans (21). Subsequently, in 2012, Antony et al. investigated 54 unrelated individuals with PCD and found 12 cases harboring biallelic variants in *CCDC39* (28). In recent years, an increasing number of studies have shown that *CCDC39*/*CCDC40* is the most frequent pathogenic gene responsible for PCD. Fassad et al. conducted a study in a multiethnic PCD cohort from 161 unrelated families and revealed that 42% of Arab families with PCD carried *CCDC39*/*CCDC40* variants (29). Similarly, an investigation conducted in Egypt suggested that 7 of 33 individuals carried biallelic variants in *CCDC39*/*CCDC40* (30). Analysis of the genetic spectrum of Chinese children with PCD showed that 5 of 51 cases harbored biallelic variants in *CCDC39* (10).

In previous studies, morphological defects in sperm were detected in male patients with *CCDC39* variants (21, 26). For example, Merveille et al. reported oligoasthenospermia combined with shortened sperm flagella in PCD patients harboring *CCDC39* variants (21). Chen et al. identified a novel homozygous variant in *CCDC39* causing PCD and MMAF. In addition, they reported that intracytoplasmic sperm injection is an effective clinical intervention for achieving positive pregnancy outcomes (26).

In this study, we identified a homozygous variant reported previously and two compound heterozygous variants of *CCDC39* in two Chinese patients with PCD and Kartagener syndrome. Furthermore, we found that the protein expression of CCDC39 was significantly decreased in sperm from the male proband (F2 II-1), who was diagnosed as having infertility due to MMAF. Using intracytoplasmic sperm injection, F2 II-1 and his wife successfully produced a child. These findings support the notion that *CCDC39* variants could be responsible for PCD, Kartagener syndrome, and MMAF.

There were several limitations to this study. First, as it is an invasive procedure, we did not collect respiratory cilia for further experiments, such as for transmission electron microscopy, high-speed video microscopy, and CCDC39 expression analysis. Additionally, both patients refused testing of the nasal nitric oxide concentration. PCD was diagnosed based on the clinical phenotype and genotyping. Second, only two families were enrolled, and no molecular epidemiologic data of *CCDC39* variants were obtained. More future studies recruiting larger cohorts are needed in order to investigate the genetic profile of patients with PCD.

## 5. Conclusion

In summary, we identified a homozygous variant and two compound heterozygous variants of *CCDC39*, which can be responsible for the pathogenesis of PCD and Kartagener syndrome in two cases, expanding the variant spectrum of PCD in Chinese patients involving *CCDC39*. And compound *CCDC39* variants can also be identified in infertile male cases with MMAF.

## Figures and Tables

**Table 1:** Clinical manifestations and semen parameters of the two PCD individuals carrying *CCDC39* variants.

Individual	F1 II-1 (female)		F2 II-1 (male)	
*PCD-related phenomenon*				
Rhinosinusitis	No			No
Wet cough	Yes			Yes
Otitis media	No			No
Bronchiectasis	Yes			Yes
Situs inversus	Yes			Yes
Congenital heart disease	No			No
*Semen analysis*				
Semen volume (mL)	/			6.7
Sperm concentration (10^6^/mL)	/			1.4
Progressive motility (%)	/			0
Normal morphology (%)	/			0
*Sperm vitality* (%)	/			NA
Sperm morphology				
Absent flagella (%)	/			3.5
Short flagella (%)	/			5.0
Coiled flagella (%)	/			82.5
Angulation (%)	/			1.5
Irregular caliber (%)	/			1.0

Abbreviations: PCD: primary ciliary dyskinesia; N/A: not available.

**Table 2:** Genetic information of *CCDC39* variants of the two PCD patients.

Subject	F1 II-1	F2 II-1	
cDNA mutation	M1: c.286C>T (homozygous)	M2: c.732_733del	M3: c.2800_2802dup
Exon	Exon 3	Exon 6	Exon20
Mutation type	Nonsense	Frameshift deletion	Nonframeshift duplication
Protein alteration	p.Arg96Ter	p.Ala245PhefsTer18	p.Val934dup
rs ID	778577109	NA	556950924
*Allele frequency in human population*			
1KGP	0	0	5.99 × 10^−4^
ExAC_all	2.72 × 10^−5^	0	1.50 × 10^−4^
gnomAD	5.16 × 10^−6^	0	1.18 × 10^−4^
*Deleterious prediction*			
SIFT	NA	NA	NA
PolyPhen-2	NA	NA	NA
MutationTaster	D	NA	NA

RefSeq accession number of *CCDC39* is NM_181426. Abbreviations: 1KGP: 1000 Genomes Project; ExAC_all: all the data of Exome Aggregation Consortium; gnomAD: Genome Aggregation Database; D: disease-causing; NA: not available.

## Data Availability

The datasets utilized in this study can be obtained on reasonable request from the authors.
